# Alpha 7 nicotinic receptors attenuate neurite development through calcium activation of calpain at the growth cone

**DOI:** 10.1371/journal.pone.0197247

**Published:** 2018-05-16

**Authors:** Justin R. King, Nadine Kabbani

**Affiliations:** School of Systems Biology, George Mason University, Fairfax, Virginia, United States of America; Weizmann Institute of Science, ISRAEL

## Abstract

The α7 nicotinic acetylcholine receptor (nAChR) is a ligand-gated ion channel that plays an important role in cellular calcium signaling contributing to synaptic development and plasticity, and is a key drug target for the treatment of neurodegenerative conditions such as Alzheimer’s disease. Here we show that α7 nAChR mediated calcium signals in differentiating PC12 cells activate the proteolytic enzyme calpain leading to spectrin breakdown, microtubule retraction, and attenuation in neurite growth. Imaging in growth cones confirms that α7 activation decreases EB3 comet motility in a calcium dependent manner as demonstrated by the ability of α7 nAChR, ryanodine, or IP_3_ receptor antagonists to block the effect of α7 nAChR on growth. α7 nAChR mediated EB3 comet motility, spectrin breakdown, and neurite growth was also inhibited by the addition of the selective calpain blocker calpeptin and attenuated by the expression of an α7 subunit unable to bind Gαq and activate calcium store release. The findings indicate that α7 nAChRs regulate cytoskeletal dynamics through local calcium signals for calpain protease activity.

## Introduction

The growth cone is a dynamic subcellular structure that guides the movement and function of growing and regenerating axons and drives synaptogenesis [1,2]. Signaling within the growth cone is driven by extracellular ligands such as growth factors and neurotransmitters, which can activate cell surface receptors. Spatio-temporal cytosolic calcium changes throughout the growth cone encode information that is vital to axon motility and growth [2–5]. Increases in local cytosolic calcium concentration can activate calmodulin-activated kinase (CaMKII), which promotes actin assembly and neurite elongation [6]. In contrast, high levels of cytosolic calcium can inhibit growth through the activation of the proteolytic enzyme calpain, which severs spectrin causing cytoskeletal breakdown at the membrane [2]. Calpains are a 15-member family of calcium-activated cysteine proteases localized to the cytosol and mitochondria with the ability to regulate a large number of substrates including proteolytic digestion of cytoskeletal proteins such as spectrin [7]. In the brain, two primary isoforms have been identified (calpain 1 and calpain 2) with differing calcium binding and effector properties [8]. PC12 cells have been shown previously to express active calpain that noticeably cleaves spectrin, making these cells a suitable system for the study of calpain function [9,10]. Cytoskeletal dynamism through calpain substrate cleavage is an important process during axon guidance, homeostatic plasticity, nerve regeneration, and post-synaptic adaptation that underlies long term plasticity in brain regions for learning and memory [10–13].

Numerous nicotinic acetylcholine receptor (nAChR) subunits are expressed in neuronal development and contribute to cholinergic signaling important for cell proliferation, survival, and synapse formation [14]. This class of ligand gated ion channel receptors is formed through an assembly of five subunits into homopentameric or heteropentameric combinations that conduct cations across the plasma membrane [15,16]. The homopentameric α7 nAChR is the second most abundant nAChR in the mammalian nervous system and maintains a high permeability to extracellular calcium [17–19]. Agonist activation of the α7 nAChR can trigger cellular calcium transients that vary in duration, amplitude, and distribution depending on the subcellular localization of the nAChR and its proximity to the endoplasmic reticulum (ER) [20–22]. α7 nAChR signaling is important for synaptogenesis and growth, functional plasticity that underlies cognition and learning, and has been implicated in the pathology of neurodevelopmental and neurodegenerative disorders [23]. The targeting of the α7 nAChR is thus appealing in drug development for major human disorders including Schizophrenia and Alzheimer’s disease (AD) [24].

In brain slices and *in vivo*, stimulation of the α7 nAChR mediates direct structural and functional changes at the synapse in regions such as dentate gyrus and CA1 of the hippocampus [25–27]. Ligand activation of the α7 nAChR in various types of cultured cells including hippocampal and cortical neurons confirms the role of α7 signaling in modulating neurite growth including elongation and branching in axons [25,28,29]. In this study, we show that α7 mediated calcium signaling at the growth cone activates the cytoskeletal regulatory enzyme calpain leading to spectrin cleavage and reduced microtubule elongation. The findings suggest that α7 nAChR calcium regulation can contribute to development and degeneration through calpain activation.

## Materials and methods

### Cell culture and transfection

Pheochromocytoma line 12 (PC12) (ATCC® CRL1721™, Gaithersburg MD, USA) cells were grown on collagen (Santa Cruz, Dallas TX, USA) (50 μg/ml) or poly-D-lysine (Millipore, Billerica, MA, USA) (100 μg/ml) matrix and differentiated by the addition of 2.5s mouse nerve growth factor (NGF) (Millipore) (200 ng/ml). Cells were cultured in RPMI media supplemented with 10% horse serum, 5% fetal bovine serum, and 1% Penicillin Streptomycin. For NGF differentiation, serum was diluted to 20% of original strength in RPMI for final concentrations of 2% horse serum, 1% fetal bovine serum, and 0.2% Penicillin Streptomycin (Thermo Fisher, Waltham, MA, USA) [29]. Cells were transfected with α7_345-348A_ [22], or red fluorescence protein tagged End-Binding Protein 3 (EB3-RFP) [20,29–31] using Lipofectamine 2000 according to the manufacturer’s instructions (ThermoFisher). Plasmid DNA was propagated in DH5A competent cells (Thermofisher) then purified using a maxi prep kit (Xymo Research, Irvine CA, USA). All vectors were sequenced using Eurofins Genomics (Louisville KY, USA). An empty vector was used as a control in experiments involving DNA transfection.

### Drug treatment

Drugs were dissolved in HBSS, which was used as the experimental vehicle, and applied directly to the media containing NGF. Drugs concentrations are based on published literature: choline (Acros Organics, Geel, Belgium) (10 mM, 3 mM, and 1 mM) [22]; α-bungarotoxin (Thermofisher) (BGTX) (50 nM) [32]; calpeptin (Sigma Aldrich, St. Louis MO, USA)(26 μM) [33], ryanodine (Santa Cruz) (30 μM) [21]; Xestospongin C. (Tocris Biosciences, Bristol, UK.) (Xest C.) (1 μM) [20]; FK506 (Tocris Biosciences) (40 μM) [34]; Substance P (Tocris Biosciences) (Sub P) (1 μM) [20]; PNU 282987 (Tocris Biosciences) (10 μM) [35].

### EB3 comet velocity and FRAP analysis

PC12 cells were transfected with the microtubule capping protein EB3-RFP in order to measure the motility and direction of the “EB3 comet” as described previously [20,30,31]. EB3 comet imaging was performed using an inverted Zeiss LSM800 confocal microscope at 63X magnification. EB3-RFP was analyzed using a 555 nm wavelength filter, and EB3 comet trajectory was followed in the growth cone using the Image J (NIH) multiple kymograph plugin. For Fluorescence Recovery After Photobleach (FRAP) analysis, 15-seconds of baseline recording was followed by photobleaching (using a 561 nm laser at 90% power for 5 seconds) of the region of interest (ROI_p_). EB3 comet recovery after photobleaching within the ROI was obtained by capturing an image every 10 seconds for 90 seconds at 2 x 2 binning. EB3 comet velocity was measured as an average of the distance traveled within the ROI over the 90s recovery period. Photo-toxicity was minimized using low-intensity and neutral density light filters as described [28]. All measurements for EB3 comet velocity are provided in S1 File. EB3 comet assay values are based on independent experiments repeated in triplicate (n = 18 cells per condition).

### Protein preparation and western blot

Membrane proteins were obtained from cultured cells as previously described [25]. Briefly, cells were lysed using a non-denaturing lysis buffer consisting of 1% Triton X-100, 137 mM NaCl, 2 mM EDTA, and 20 mM Tris HCl (pH 8) supplemented with protease and phosphatase inhibitors (Roche, Penzeberg, Germany). Protein concentration was determined using a Bradford protein assay kit (Thermo Fisher). Proteins were separated on Bis-Tris gradient gels, and transferred onto nitrocellulose (ThermoFisher). Primary antibodies used were anti: αIIspectrin (Santa Cruz), and GAPDH (Cell Signaling, Danvers MA, USA). HRP secondary antibodies were purchased from Jackson Immunoresearch (West Grove PA, USA). A SeeBlue Ladder (ThermoFisher) was used as a protein standard. Bands were visualized using SuperSignal West Pico Chemiluminescent substrate (ThermoFisher) via the G:BOX Imaging System and GeneSYS software (Syngene, Fredrick MD, USA). Band density analysis was performed in Image J (NIH, Bethesda MD, USA). All measures were normalized to GAPDH controls. Protein measures in the study are based on averages from three independent experiments (n = 3).

Spectrin breakdown assays were performed as previously described [10]. Briefly, cells were pre-treated with inhibitors (calpeptin (26 μM), BGTX (50 nM), or Sub P (1 μM)) for 1 hour prior to the agonist application (choline (10 mM) or PNU 282987 (10 μM)) for 2 hours before cell lysis and processing as described above. Control cells were treated with the vehicle only under similar experimental conditions.

### Cell viability

Cell viability was measured using a WST-1 Cell assay (Cayman Chemical Company, Ann Arbor, MI, USA). Cells were plated in a 96 well dish and differentiated in the presence of NGF and drug treatments as done in prior experiments. WST-1 dye was added to cells for 2 hours. Cells were then imaged using a Varioskan Flash (Thermo Scientific) plate reader. The WST-1 signal was measured using a 405 nm laser, and experiments are representative of 6 measures per group. To control for background, a group of cells was treated with ethanol for 5 minutes to kill all cells prior to the addition of the dye. The average value of these wells was then subtracted from averages to correct for background fluorescence caused by the dye.

### Immunocytochemistry and fluorescence imaging

PC12 cells were fixed in a solution consisting of 1x PEM (80 mM PIPES, 5 mM EGTA, and 1 mM MgCl_2_, pH 6.8) and 0.3% glutaraldehyde. Cells were permeablized by the addition of 0.05% Triton X-100 (Sigma Aldrich) [36]. Sodium borohydride (2 mg/ml) was used for glutaraldehyde quenching. Cells were labeled with rhodamine phalloidin (Cytoskeleton, Denver CO, USA) in order to visualize F-actin and the structural contour. Images were captured using an inverted Zeiss LSM800 confocal microscope and with the Zen software package (Carl Zeiss AG, Oberkochen, Germany). Morphometric measures were carried out in ImageJ (NIH, Bethesda, MD, USA). Neurites longer than the soma (>10 μm) were reconstructed using the Vaa3D software [37,38] (n = 20 reconstructions per condition). All reconstructed SWC files were deposited in the public repository, Neuromorpho.org an open source database for neuronal reconstructions. All raw data measurements for cell growth of both WT and α7_345-348A_ expressing PC12 cells are provided in S2 File. Growth cones were identified based on the criteria described in [39].

### Statistical analysis

Data are shown as mean ± SEM and are representative of at least three independent experiments in each assay. Statistical analysis was performed using a one-way analysis of variance (ANOVA) to determine significance between mean values. All groups showed a normal distribution, and passed Levene’s test of homogeneity before ANOVA’s were run. Tukey HSD post-hoc tests were used for individual group comparisons where appropriate. A minimum statistical value p<0.05 was considered significant.

## Results

### Ligand activation of α7 nAChRs leads to neurite retraction and growth cone collapse

**Fig 1:**
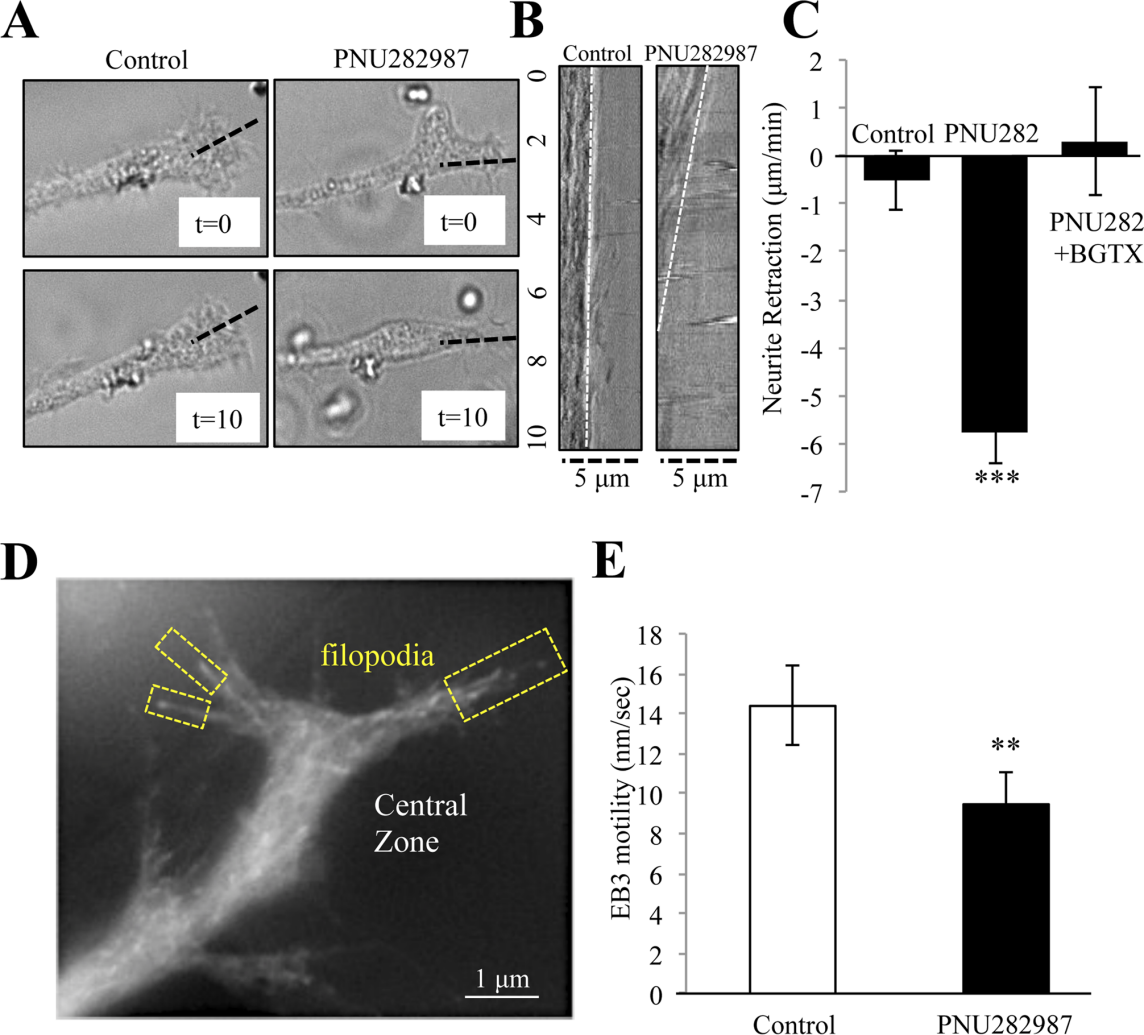
α7 nAChRs activation drives to neurite retraction and inhibits microtubule growth. Brightfield imaging of the neurite and its growth cone in NGF differentiated PC12 cells treated with Control (DMSO) or 10 μM of the α7 nAChR specific agonist PNU282987 for 10 min. (A) Representative images of the growth cone at the beginning of the experiment (t = 0) and at the end of the experiment (t = 10). (B) Kymographs of the dotted line in (A) showing drug effect on neurite retraction during the experiment. (C) Average rate of neurite retraction for each group. (D) A representative image of EB3-RFP (EB3 comet) expression in the growth cone. (E) EB3 comet velocity in the growth cone following drug application. (*** = p<0.001; n = 10 cells per group).

α7 nAChRs are expressed during development and contribute to cholinergic mechanisms of growth and plasticity in the central nervous system [40,41]. In various cell types, including cultures of primary neurons and differentiating PC12 cells, pharmacological activation of the α7 nAChR has been shown to alter neurite elongation and branching [25,28,29]. We have previously shown that α7 nAChRs are abundant within the growth cone and that activation of the α7 nAChR in hippocampal neurons with selective agonists such as PNU282987 or choline can attenuate growth [20,29]. We tested the effect of α7 nAChR activation on neurite elongation in differentiating PC12 cells using brightfield time-lapse imaging. PNU282987 treatment (10 αM) was associated with neurite retraction that can be measured from the tip of the growth cone. As shown in Fig 1A and 1B, at 10 min application of PNU282987 the primary neurite had undergone a noticeable retraction. A kymograph rendering of the retracting neurite confirms that drug application is associated with a retrograde movement from the tip of the growth cone (Fig 1B). In addition, live imaging experiments (Fig 1C), indicate that after a 10 min application of PNU282987 the growth cone compartment is noticeably smaller suggestive of growth cone collapse due to drug treatment This effect was blocked by pre-treatment with the specific α7 nAChR antagonist α-bungarotoxin (BGTX), restoring movement of the growth cone back to control levels (Fig 1C). These experiments suggest that pharmacological activation of the α7 nAChR promotes rapid neurite retraction through retrograde steering of the growth cone and that this process may contribute to the ability of the α7 nAChR to promote long-term changes in axon growth.

### α7 nAChR activation inhibits microtubule entry into the growth cone

α7 nAChRs are targeted to the growth cone in PC12 cells, hippocampal neurons, and are detected within growth cone fractions of the embryonic rodent brain [22,25]. In the growth cone, α7 nAChRs localize to the central zone region that houses a microtubule protein network [20]. We have shown that activation of the α7 nAChR in the growth cone mediates calcium influx into the cell through the opening of the α7 nAChR channel and the mobilization of intracellular calcium release from local ER through both calcium induced calcium release (CICR) and inositol induced calcium store release (IICR) [20]. To determine if α7 nAChR activation regulates microtubule growth, cells were transfected with an RFP tagged EB3 microtubule capping protein (EB3-RFP or EB3 comets) in order to measure the movement of the microtubule “plus” end in individual filopodia of the growth cone [30]. As shown in Fig 1D and 1E, α7 nAChR activation with PNU282987 (10 μM) slows EB3 comet movement into the growth cone. These findings are consistent with earlier studies showing that α7 nAChR activation mediates neurite retraction from the growth cone and possibly through receptor driven calcium.

### EB3 comet motility in the growth cone is controlled by α7 nAChR calcium signaling

**Fig 2:**
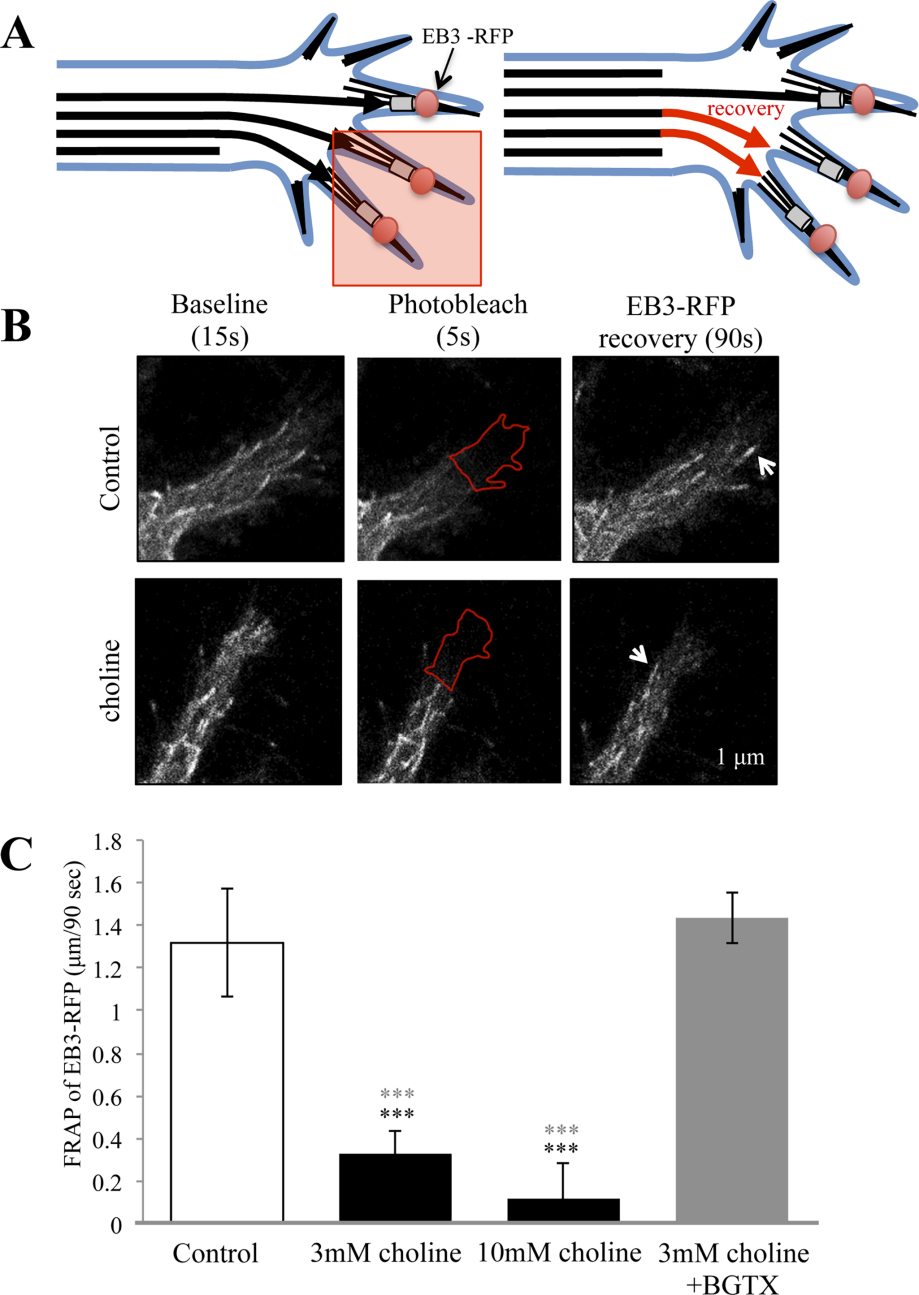
The α7 nAChR directly attenuates in EB3 entry into the growth cone. (A) A schematic showing the fluorescence recovery after photobleaching (FRAP) assay. The red box indicates the photobleached area of the growth cone, while the red arrows represent EB3 comet re-entry into the bleached area during recovery. (B) Representative images of EB3 comets in the growth cone under Control (HBSS) and choline (3mM) treated condition during the experiment. Arrows point to the location of EB3 comets recovered after photo-bleaching in the ROI indicated by the red outline. (C) Average distance traveled by EB3 comets following photo-bleaching in Control, choline treated, and choline treated following BGTX application in cells. (*** = p<0.001; Black asterisks indicate comparison to control, Grey asterisks indicate comparison to 3 mM choline+BGTX treatment; n = 18 cells per group).

We tested the ability of the α7 nAChR to regulate microtubule dynamics in the growth cone using a fluorescence recovery after photo-bleaching (FRAP) method (Fig 2A). In these experiments the EB3-RFP signal was measured within the photo-bleached ROI (ROI_p_) allowing us to calculate the rate of microtubule re-entry under varying experimental conditions. Fig 2B shows representative images from control and drug treated cells before, immediately after, and 90 seconds following the recovery from photo-bleaching. In control cells treated with the vehicle, EB3 re-entry within the ROI_p_ averaged 1.32 μm over 90-seconds (0.0145 μm/sec) (Fig 2C). Under conditions of α7 activation with choline, EB3 re-entry within the ROI_p_ was significantly attenuated (ANOVA: F (3,70) = 15.297, p<0.001) to 0.33 μm/90 sec (0.0036 μm/sec) (Post Hoc:p<0.001 to control) and 0.12 μm/90 sec (Post Hoc:p<0.001 to control) as function of 3 mM and 10 mM choline treatment, respectively (Fig 2C). An analysis of the ROI_p_ region in choline treated cells indicates that at 90 seconds of recovery the average size of the ROI_p_ is not altered from the starting point of the experiment. Pre-incubation with the specific α7 nAChR antagonist BGTX was able to block the ability of choline to influence EB3 motility in the cell (1.43 μm over 90-seconds; 0.0159 μm/sec) (Fig 2C).

**Fig 3:**
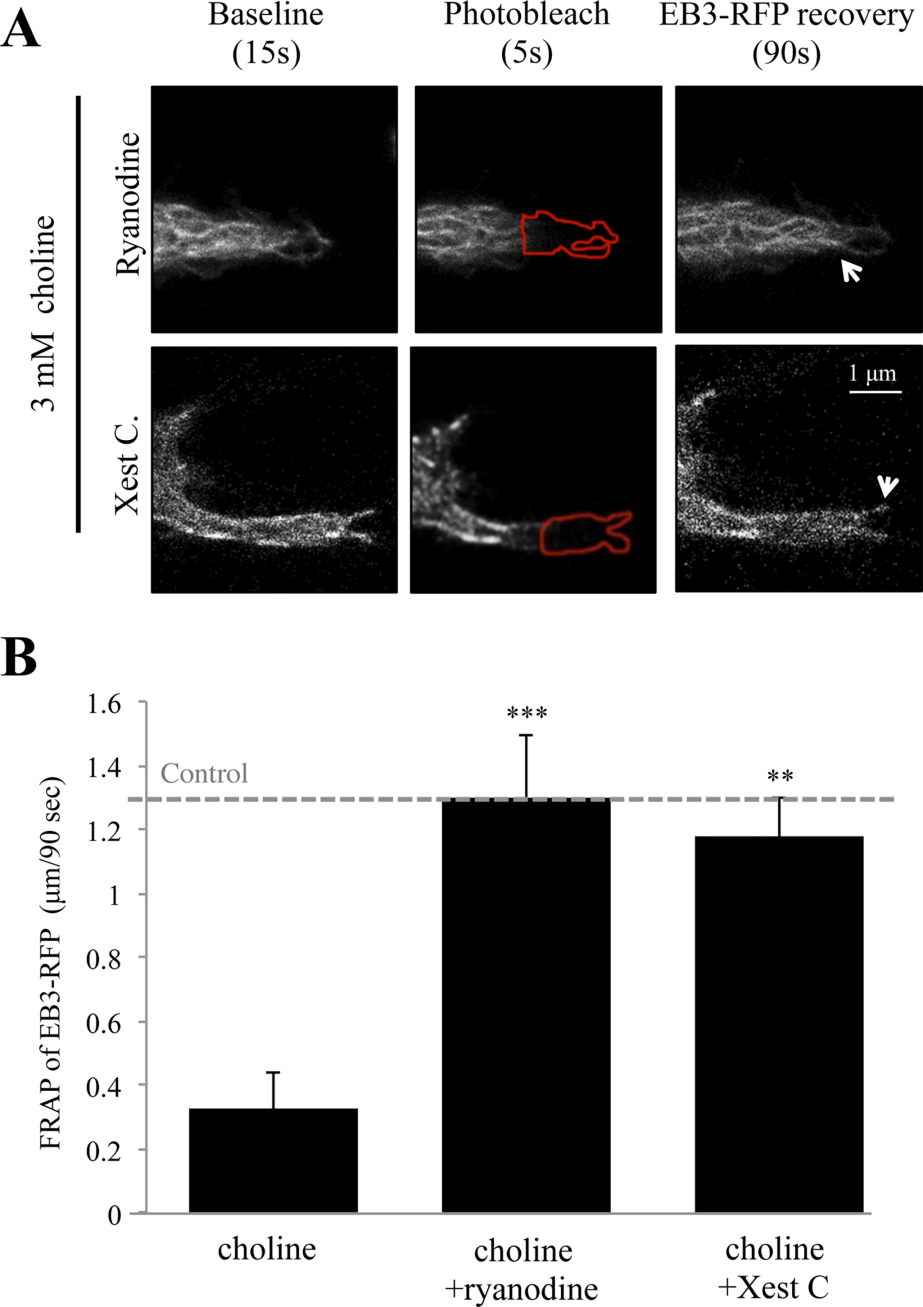
Intracellular calcium store release participates in choline mediated EB3 motility in the growth cone. (A) Representative images of choline mediated EB3 comet motility in ryanodine (30 μM) and Xest C (1 μM) pre-treated cells during the experiment. Arrows point to the location of EB3 comets recovered after photo-bleaching in the ROI indicated by a red outline. (B) Average distance traveled by EB3 comets following photo-bleaching in choline (3 mM) treated cells that were pre-incubated with 30 μM ryanodine or 1 μM Xest C. Control: EB3 comet velocity in non-treated cells. (** = p<0.005, *** = p<0.001; n = 18 cells per group).

Studies have shown that free intracellular calcium in growth cones is an important regulator of turning, retraction, and extension [42]. Calcium fluctuations within the growth cone are also important for structural elongation, which is essential during axon pathfinding *in vivo* [43]. We have shown that α7 nAChR activation increases intracellular calcium levels within the growth cone of differentiated PC12 cells through ryanodine and IP_3_R associated CICR and IICR, respectively [22]. To test the role of intracellular calcium on EB3 comet velocity, cells were pre-incubated with the ryanodine receptor blocker ryanodine (30 μM) or the IP_3_R inhibitor Xest C (1 μM) for 30 mins prior to the FRAP assay. As shown in Fig 3, pre-incubation with ryanodine was associated with a decrease in the ability of choline to influence EB3 movement within the ROI_p_. Specifically, in cells treated with ryanodine the average EB3 comet velocity was 1.30 μm/90 sec (0.0144 μm/sec), which represents a complete recovery to the control baseline condition (ANOVA: F (2,53) = 19.16, p<0.001; Post Hoc:p<0.001 compared to choline 3 mM) (Fig 3B). Similarly, pre-treatment with Xest C diminished the effect of choline on EB3 re-entry into the ROI_p_ with average EB3 comet rates at 1.18 μm/90 sec (0.0131 μm/sec) (Post Hoc: p = 0.002 compared to choline 3 mM) (Fig 3B). These findings indicate that α7 nAChR calcium signaling through the ER is necessary for choline regulation of microtubule growth in the growth cone.

### α7 nAChR calcium signaling activates calpain in the growth cone

**Fig 4:**
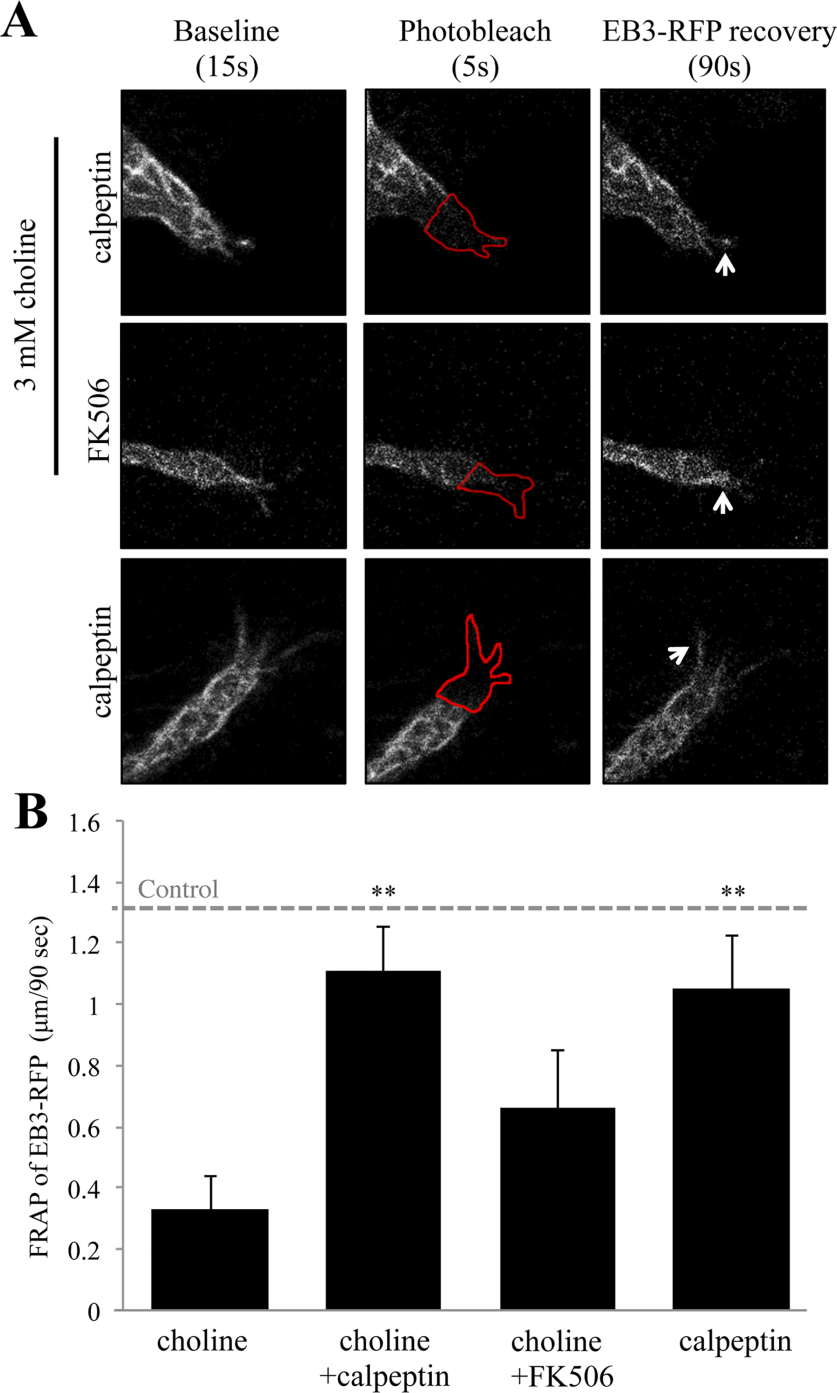
Inhibition of calpain but not calcineurin abolishes choline regulation of EB3 comet velocity. (A) Representative images of choline (3 mM) mediated EB3 comet motility in calpeptin (26 μM) and FK506 (40 μM) pre-treated cells during the experiment. Arrows point to the location of EB3 comets after photo-bleaching in the ROI indicated by a red outline. (B) Average distance traveled by EB3 comets following photo-bleaching in treated cells relative to Control: EB3 comet velocity in non-treated cells. (** = p<0.005; n = 18 cells per group).

Calcium transients can regulate growth cone motility through cytoskeletal regulatory elements such as the protease calpain, which severs actin networks through spectrin cleavage [44]. We tested the involvement of calpain in α7 mediated microtubule entry into the growth cone. In these experiments, differentiated PC12 cells were pre-incubated with the calpain inhibitor calpeptin (26 μM) prior to FRAP analysis of EB3-RFP re-entry. As shown in Fig 4, calpeptin was associated with a loss in the ability of choline (3 mM) to influence EB3 re-entry into the ROI_p_. In cells pre-treated with calpeptin, choline application was associated with EB3 comet rates of 1.11 μm/90 sec (0.0123 μm/sec) in comparison to EB3 comet rates of 0.33 μm/90 sec (0.0036 μm/sec) obtained through choline treatment alone (ANOVA: F (3,71) = 5.748, p = 0.001; Post Hoc:p = 0.004 compared to choline 3 mM) (Fig 4). Pre-treatment of cells with calpeptin alone showed a similar response to those cells treated with choline 3 mM after calpeptin pre-incubation (1.07 μm/90 sec; 0.0119 μm/sec; Post Hoc between the control and calpeptin alone treated cells: p = 0.998) (Fig 4B).

Ligand-induced calcium signaling can also lead to growth cone collapse via the activity of the calcium sensitive protein phosphatase calcineurin (PP2B) [45]. We have previously shown a role for calcineurin in nicotine-mediated neurite growth in PC12 cells [25]. We thus tested the possibility that α7 nAChR regulation of microtubule motility in the growth cone involves calcium signaling through calcineurin. Cells were pre-treated with the calcineurin inhibitor FK506 (40 μm) for 30 minutes prior to the FRAP assay. Interestingly, FK506 did not significantly alter the rate of choline (3mM) mediated EB3 re-entry into the ROI_p_ (Fig 4B). Specifically, in cells pre-treated with FK506, choline treatment was associated with an average EB3 re-entry rate of 0.48 μm/90 sec (0.0053 μm/sec), which was found to be not statistically significant from choline treatment alone (Post Hoc:p = 0.272 compared to choline 3 mM) (Fig 4). The data indicates that calpain, but not calcineurin, is activated by α7 nAChR calcium signaling in the growth cone.

### α7 nAChR activated calpain promotes spectrin breakdown

**Fig 5:**
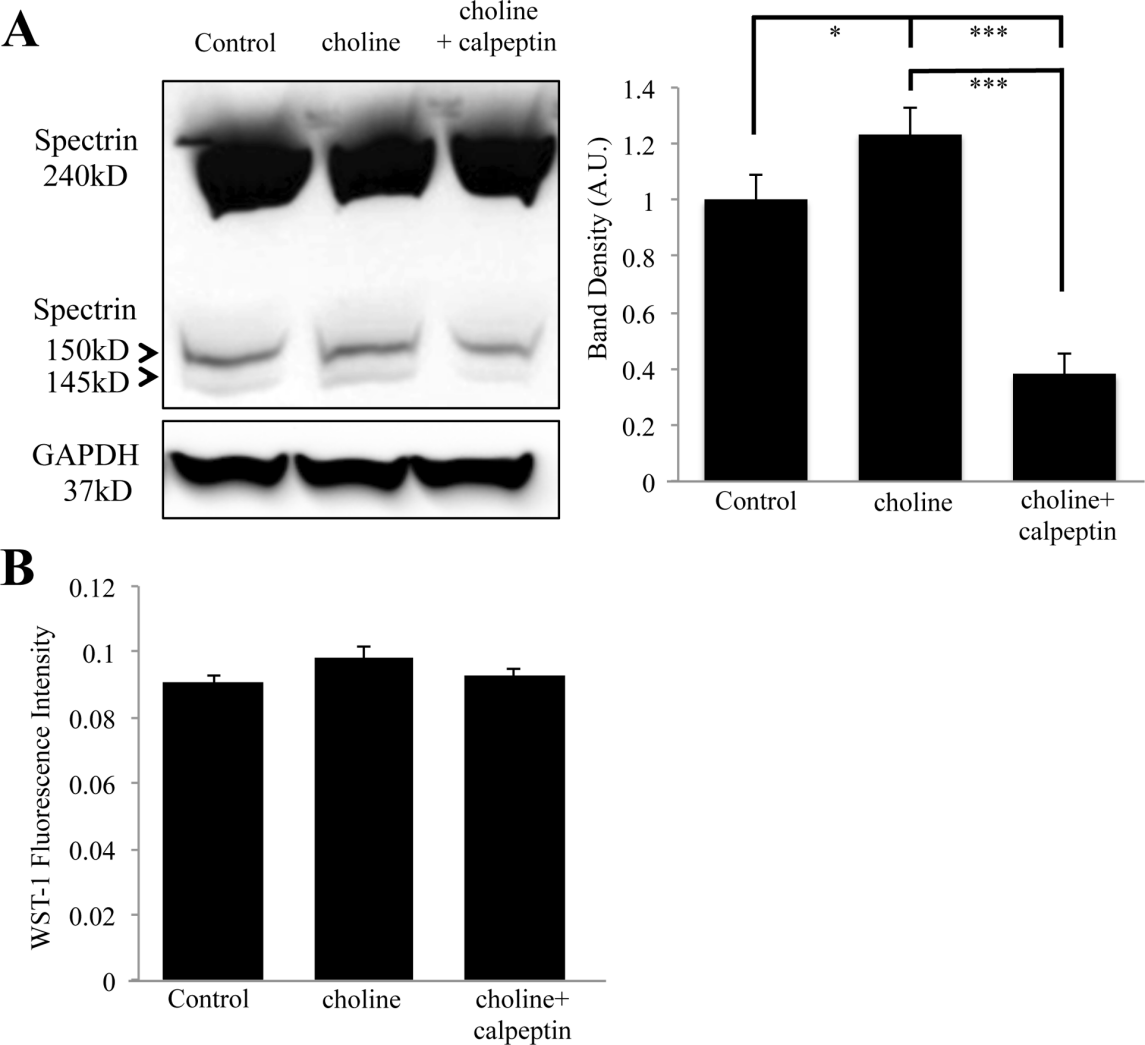
Choline mediated spectrin breakdown is inhibited by calpeptin. (A) Left, a western blot showing anti-αIIspectrin immunoreactive bands at 240 kD, 150 kD, and 145 kD. Lanes were loaded with lysates from PC12 cells treated with 10 mM choline, 26 μM calpeptin and choline, or HBSS (Control). Right, band density analysis from three independent experiments showing an effect of choline on the αIIspectrin breakdown. Band density was calculated by combining the density of the 150 kD and the 145 kD spectrin breakdown bands. (B) WST-1 fluorescence showing cell viability during drug treatments (* = p<0.05, *** = p<0.001; n = 3 independent experiments).

Calcium activation of calpain has been shown to drive axon elongation and regeneration after injury through proteolytic breakdown of the submembrane spectrin network [11,46]. Calpain mediated degradation of αIIspectrin results in the formation of two stable breakdown products that migrate at 150 kDa and 145 kDa on a western blot [8,47]. This breakdown of αIIspectrin differs from caspase activity seen in apoptotic pathways, which leads to a different breakdown profile of spectrin [10]. We tested the ability of choline to regulate spectrin breakdown in differentiating PC12 cells under the same conditions required for neurite retraction and EB3 retention. As shown in Fig 5, western blot detection confirms the presence of two anti-spectrin immunoreactive protein bands at the molecular weights of 145 kD and 150 kD, and a full spectrin band at 240 kD. The αIIspectrin breakdown products were differentially regulated by choline treatment. Specifically, a two-hour application of 10 mM choline was associated with a noticeable increase in the αIIspectrin breakdown products in the assay (Fig 5 and Table 1). In cells pre-treated with calpeptin (26 μM for 1 hour), choline exposure was not associated with a significant production of the αIIspectrin breakdown products (Fig 5 and Table 1) suggesting that calpain activity is necessary for choline mediated spectrin breakdown. Band density analysis from three independent experiments confirms significance between control, choline, and choline+calpeptin treatment conditions (ANOVA: F (2,16) = 34.770, p<0.001; Post Hoc: p = 0.023 compared to control) and choline+calpeptin co-application (Post Hoc:p = 0.010) to both control and choline treatment alone (Fig 5). As shown, the full-length spectrin bands remain relatively unchanged (Fig 5). To ensure that drug treatment does not effect cell viability, a live cell viability assay was conducted under similar treatments conditions. As shown in Fig 5B, drug treatment was not associated with a change in cell health in this assay.

**Table 1:** Band density analysis of αIIspectrin breakdown based on percent change from the vehicle treated control.

Treatment	150 kD Band	145 kD Band
Choline (3 mM)	23.24+/-4.22%	24.52+/-9.52%
Choline + Calpeptin (26 μM)	-68.13+/-6.34%	-72.72+/-12.80%
Choline + BGTX (50 nM)	-16.10+/-5.84%	-13.79+/-3.4%%
Choline + Substance P (1 μM)	-30.15+/-13.4%	-27.36+/-7.49%
PNU282987 (10 μM)	30.54+/-3.25%	28.18+/-5.94%

**Fig 6:**
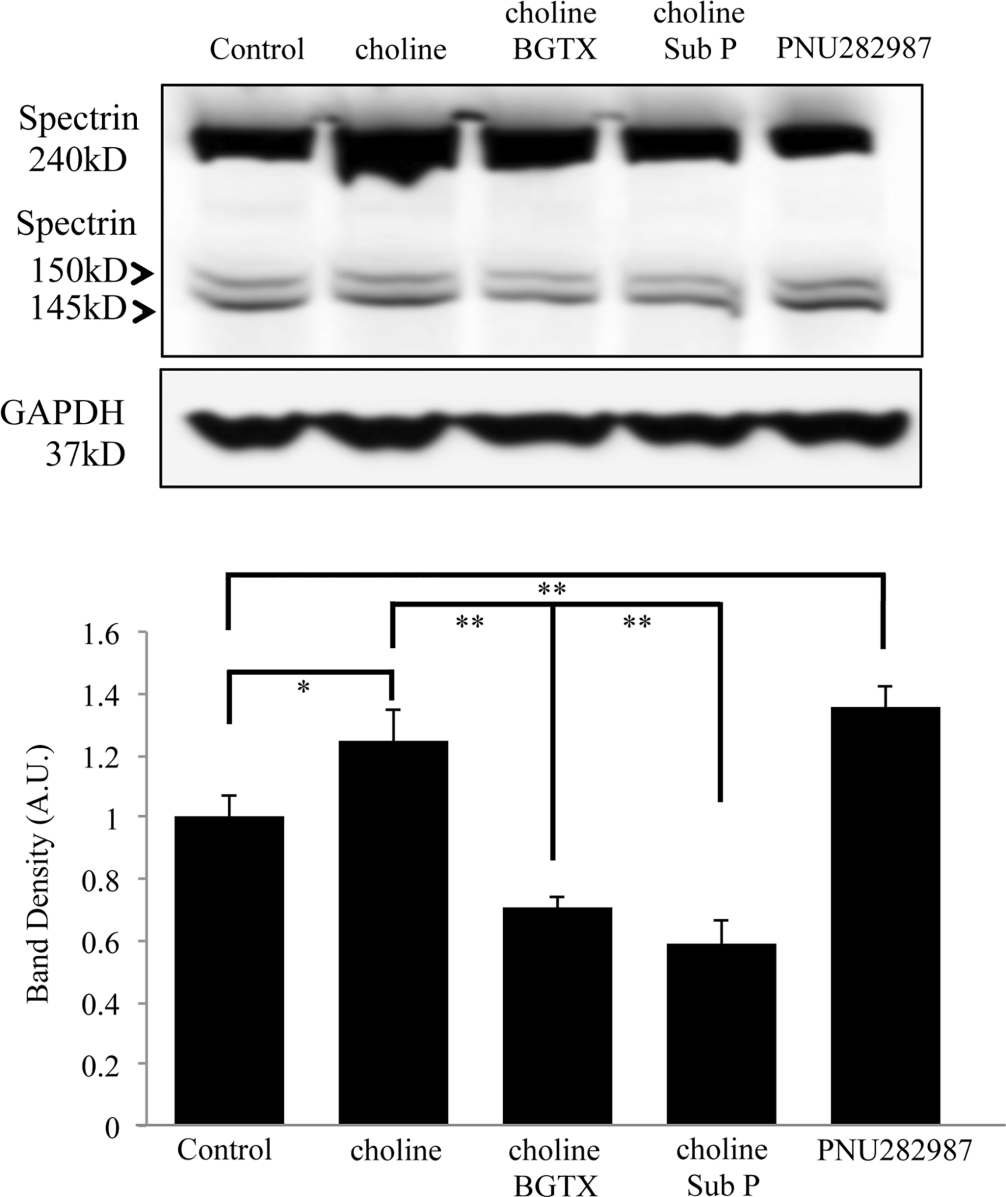
Pharmacological α7 nAChR activation mediates spectrin breakdown. Top, a western blot showing anti-αIIspectrin immunoreactive bands at 240 kD, 150 kD, and 145 kD. Lanes were loaded with lysates from PC12 cells treated with choline (10 mM) (choline), choline 10 mM) with BGTX (50 nM) or Sub P (1 μM), or PNU282987 (10 μM). Vehicle (HBSS) was used as the Control. Bottom, band density analysis from four independent experiments showing an effect of drug treatment on αIIspectrin breakdown. (* = p<0.05** = p<0.005, *** = p<0.001; n = 4 independent experiments).

We examined the ability of the α7 nAChR specific inhibitor BGTX and the Gαq inhibitor Substance P (Sub P) to block calpain mediated cleavage of αIIspectrin. These compounds were chosen in order to confirm that α7 nAChR channel activity (BGTX) and G protein coupled calcium store release (Sub P) are essential for α7 nAChR activation of calpain [22]. The specific α7 nAChR agonist PNU282987 was also tested in order to confirm the specificity α7 nAChRs in mediating spectrin breakdown. As shown in Fig 6 and Table 1, treatment of cells with BGTX or Sub P was associated with a significant decrease (ANOVA: F (4,19) = 22.440, p<0.001) in choline mediated αIIspectrin breakdown (Post Hoc: BGTX:p = 0.001 compared to choline treatment alone; Post Hoc: Sub P:p<0.001 compared to choline treatment alone). Treatment with BGTX or Sub P appeared to decrease the levels of αIIspectrin breakdown to lower than the control condition. This effect however was not found to be statistically significant. Treatment of cells with the α7 specific agonist PNU282987 (10 μM) was associated with a significant increase in the level of the αIIspectrin breakdown product (Post Hoc PNU282987:p = 0.018 to control; p<0.001 compared to both choline+BGTX and choline+Sub P) (Fig 6 and Table 1). The data confirms that α7 nAChR activation contributes to rapid cytoskeletal remodeling through calpain function.

### Inhibiting calpain boosts neurite growth in differentiating cells

**Fig 7:**
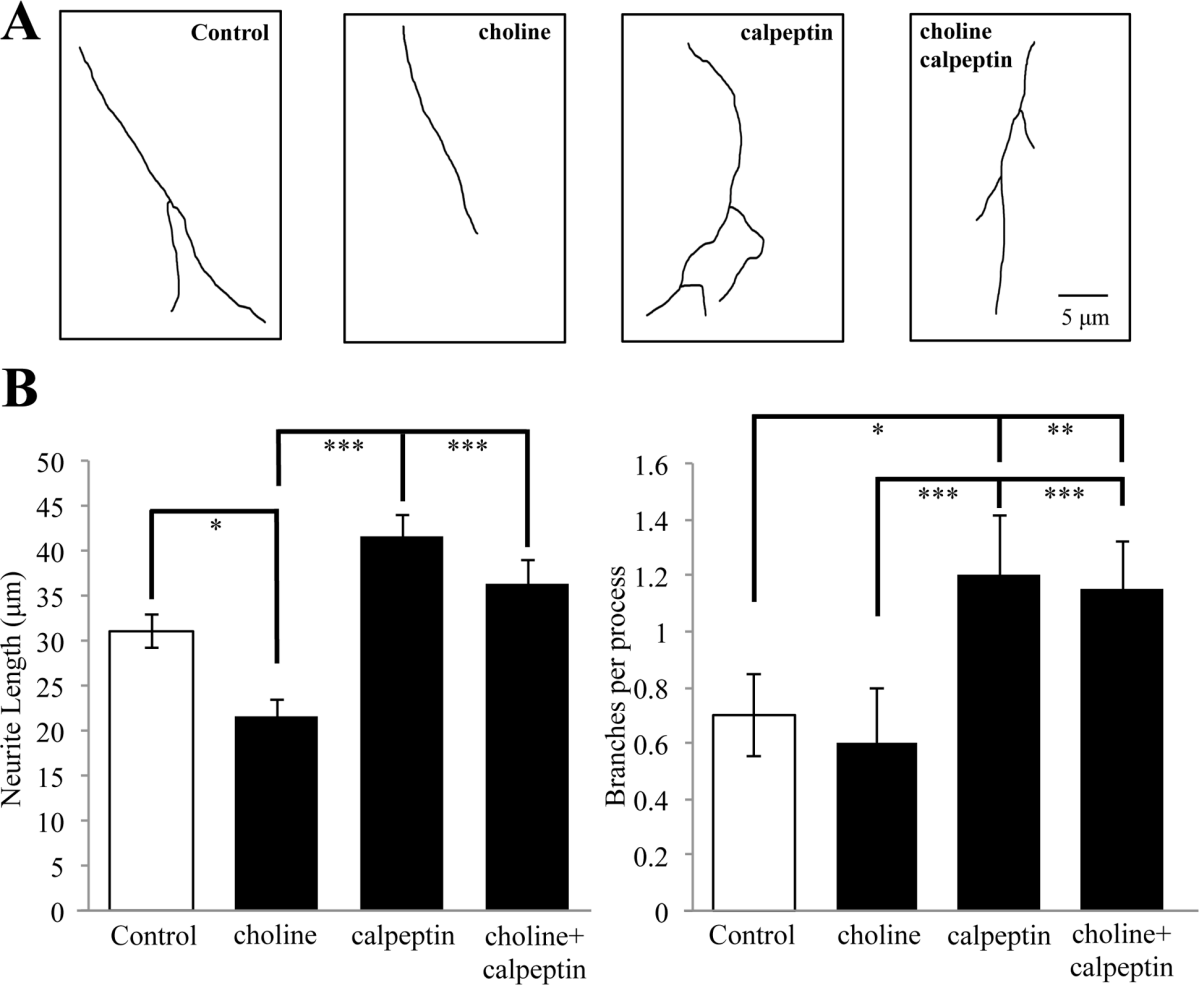
α7 nAChRs regulate neurite outgrowth through calpain. (A) Representative traces of primary neurites in PC12 cells differentiated with NGF for 3 days in the presence of HBSS (Control), 1 mM choline, 26 μM calpeptin, or choline and calpeptin. (B) Average neurite length and branching in drug treated cells (* = p<0.05,** = p<0.005, *** = p<0.001; n = 20 cells per group).

We have shown that sustained activation of the α7 nAChR leads to a decrease in the overall length and branching of neurites in PC12 cells, an effect also seen in axons of cultured hippocampal and cortical neurons [20,25,29]. We tested the involvement of calpain in α7 mediated neurite elongation and branching in PC12 cells. Cells were differentiated with nerve growth factor (NGF) for 3 days in the presence of choline (1 mM), calpeptin (26 μM), or choline and calpeptin together prior to fixation and morphological analysis. Control cells were treated with the vehicle (HBSS) under similar conditions. As shown in Fig 7, choline treatment is associated with a significant decrease in neurite length relative to controls (control: 31.03 μm vs. choline: 21.58 μm, respectively)(ANOVA: F (3,75) = 13.182, p<0.001; Post Hoc: p = 0.028 compared to control). This effect of choline was inhibited by the presence calpeptin, which restored neurite length to the levels seen in controls (36.45 μm) (Post Hoc: p = 0.313 compared to control) (Fig 7). In this experiment, treatment with calpeptin alone enhanced neurite outgrowth (41.53 μm) (Post Hoc: p = 0.007 compared to control), consistent with evidence that endogenous calpain activity contributes to neurite development [33]. Similar findings were observed when we examined the branching of the primary neurite. As shown in Fig 7, at 3 days of differentiation the application of calpeptin either alone or in conjunction with choline was associated with a significant increase in branching (ANOVA: F (3,75) = 10.152, p<0.001). The findings are consistent with experiments on the acute actions of calpain in α7 nAChR mediated cytoskeletal changes in the growth cone, suggesting α7/calpain interactions contribute to long-term structural changes in cells.

### Metabotropic α7 nAChR signaling is also required for calpain activity

**Fig 8:**
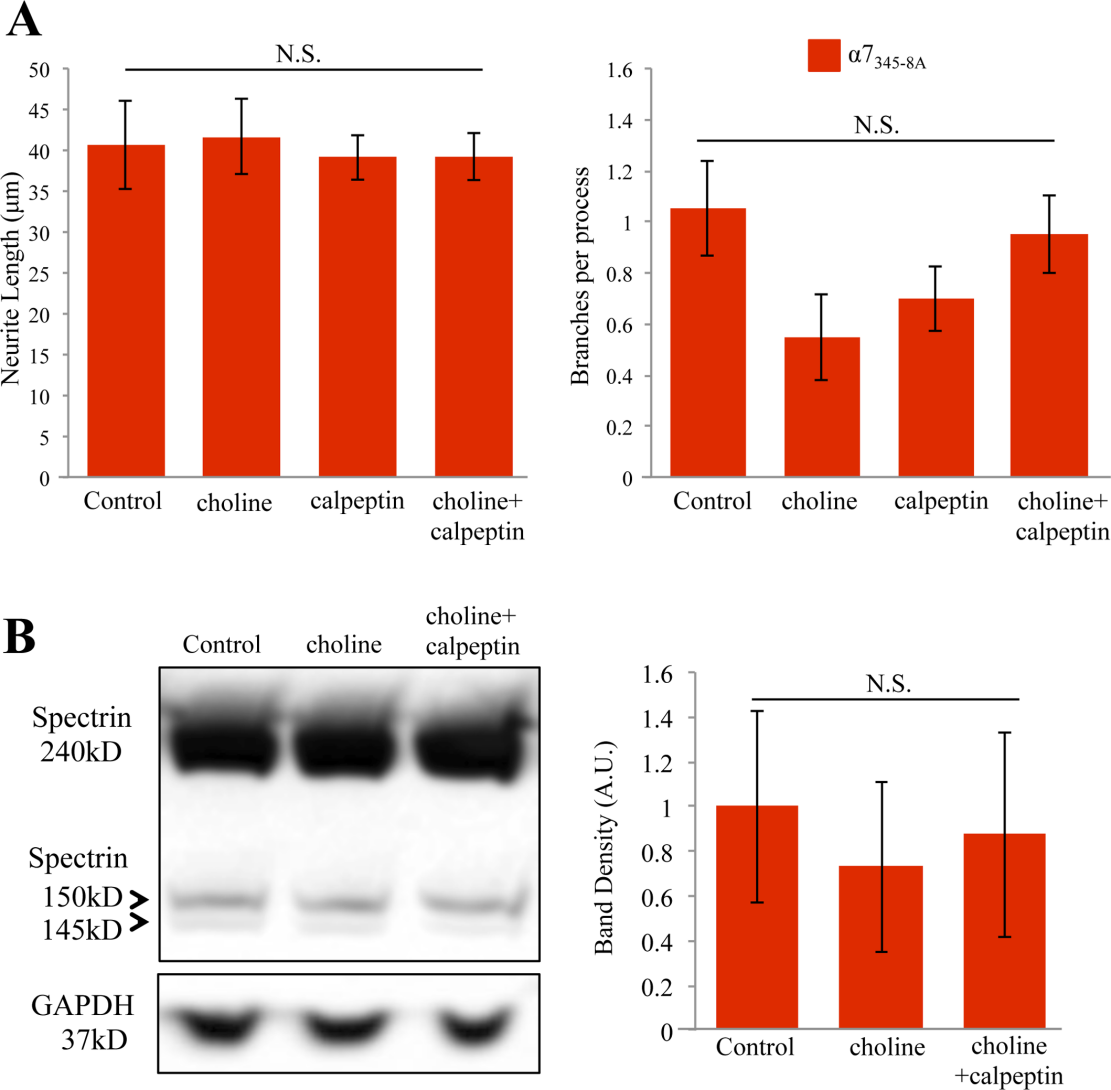
G protein coupling to the α7 nAChR is necessary for calpain mediated cytoskeletal growth. (A) Average neurite length and branching in cells transected with α7_345-8A_ at 3 days of NGF differentiation. Cells were treated with HBSS (Control), 1 mM choline, 26 μM calpeptin, or choline and calpeptin together. (B) Left, a western blot showing anti-αIIspectrin immunoreactive bands at 240 kD, 150 kD and 145 kD. Lanes were loaded with lysates from PC12 cells treated with 10 mM choline, 26 μM calpeptin then choline, or HBSS (Control). Right, band density analysis from 3 independent experiments showing the effect of drug treatment on αIIspectrin breakdown. (N.S. not significant; n = 20 cells per group for morphometric data; n = 3 independent experiments for western blot).

α7 nAChR-mediated calcium signaling occurs via ionotropic calcium influx due to the opening of the α7 channel and metabotropic activity through G protein coupling [22,48]. The later is associated with IICR and has been shown to regulate neurite outgrowth in PC12 cells [20,29]. We assessed the involvement of G protein binding in α7 nAChR mediated calpain activity and its effect on neurite outgrowth by transfecting cells with a dominant negative α7 subunit (α7_345-8A_) shown to be impaired in Gαq signaling and calcium store release [22]. This plasmid shows a transfection efficiency of 75% in PC12 cells, and has been shown to abolish the effects of choline on endogenous α7 mediated calcium signaling [22]. In this study, transfection with α7_345-8A_ was found to slightly increase neurite length and branching in differentiating PC12 cells as previously reported, although this increase was not found to be statistically significant (Fig 8A) [29]. Cells transfected with the α7_345-8A_ subunit and treated with the vehicle solvent (HBSS) were used as the control condition. Expression of the α7_345-8A_ subunit was associated with a loss in the ability of choline to impact neurite growth when compared to controls (Fig 8A). Interestingly, the effect of calpeptin on neurite growth was also lost in cells expressing the α7_345-8A_ subunit (ANOVA: F (3,79) = 0.294 p = 0.830) (Fig 8A). The effect of choline on the branching of the primary neurite was similar in this study. As shown in Fig 8A, choline treatment was not associated with a significant decrease in neurite branching even in cells that express the α7_345-8A_ subunit. The application of calpeptin either alone or with choline did not show a significant effect on branching. An ANOVA confirms that α7_345-8A_ subunit abolishes the effects of choline and calpain in this assay (ANOVA: F (3,79) = 2.035, p = 0.116).

To confirm involvement of metabotropic α7 nAChR signaling through G proteins in calpain mediated spectrin breakdown, we transfected PC12 cells with α7_345-8A_ and repeated the spectrin assay. Western blot detection indicates a loss in the ability of choline to produce the αIIspectrin breakdown product when the α7_345-8A_ subunit is present in the cell (Fig 8B and Table 2). In addition, pre-incubation with calpeptin also had no effect on αIIspectrin breakdown product levels when the α7_345-8A_ subunit is present in the cell (Fig 8B and Table 2). Band density analysis of independent experimental measures of the αIIspectrin breakdown product statistically confirms that α7_345-8A_ subunit expression is associated with a loss in choline-mediated calpain activity in this assay (ANOVA: F (2,11) = 0.101, p = 0.905) (Fig 8B).

**Table 2:** Band density analysis of αIIspectrin breakdown in cells transfected with the α7_345-8A_ subunit based on percent change from α7_345-8A_ transfected controls.

Treatment	150 kD Band	145 kD Band
Choline (3 mM)	-18.92+/-8.31%	-23.59+/-8.80%
Choline + Calpeptin (26 μM)	-11.45+/-5.40%	-12.25+/-14.17%

## Discussion

### Calcium activation of calpain in development and disease

A number of cellular systems including differentiated PC12 cells, sympathetic, cortical, and hippocampal neuronal cultures are useful for the study of neurite development through growth cone function. Findings from such work enables an understanding of key principles that guide synaptic development and can be applied to advancing treatment for nerve regeneration [49–51]. Results presented in this study suggest that α7 nAChR calcium signaling can contribute to neuro regenerative processes through calpain activity. This process appears dependent on the ability of the α7 nAChRs to foster a strong rise in intracellular calcium levels locally through coupling to the ER. This notion is consistent with the calcium driven growth cone model originally proposed by Kater and Mills [2] and expanded by others [52]. In this framework intracellular calcium signals (levels, duration, and distribution) regulate cytoskeletal elements and membrane dynamics leading to neurite elongation or retraction and turning of the growth cone. Studies in rodents and in cultured cells support this and confirm that high intracellular calcium levels in axons participate in proper synaptic pruning in development but may drive long-term damage in adulthood. To this end, pharmacological blockade of calcium channels during axon recovery in rodent models of axon injury promote survivability via a decrease in calpain activity [13,53]. Thus calcium driven calpain activity may contribute to various forms of pathogenesis including AD and traumatic brain injury [54].

How calcium signaling through calpain impacts neuronal growth and function in the context of development or aging is neither entirely clear nor simple. Our findings confirm a role for endogenous calpain activity in neurite development during differentiation [33] as confirmed by our findings that addition of calpeptin alone can increase neurite length through a decrease in spectrin breakdown relative to the baseline differentiation state. Interestingly, the addition of calpeptin alone did not appear to impact EB3 comet velocity in the growth cone. However, when combined with choline, calpeptin was able to inhibit the effect of choline on microtubule entry into the growth cone suggesting that α7 nAChR regulate cytoskeletal growth through calpain.

Studies on retinal ganglion cell degeneration suggest that calpain isoforms, which markedly differ in their calcium sensitivity and effector targets, have opposing effects on regenerative cell health with calpain 1 driving survival and calpain 2 promoting cell death [55]. Here, we find that α7 nAChR activation of calpain attenuates neurite growth through cytoskeletal disassembly at the growth cone. Whether this process in PC12 cells is mediated by calpain 1, calpain 2, or both is not clear since experimental probes of calpain function, including inhibitors such as calpeptin, do not distinguish between the two isoforms. Since cells express both types of calpain, it is plausible that different calpain isoforms participate in the signaling effects of the α7 nAChR during growth. This may explain differences in the effects of calpeptin on short *vs*. long-term neurite and cytoskeletal growth. In future studies it will be interesting to determine if α7 nAChR activation leads to the activity of calpain 1, 2, or both and if this is cell type and compartment specific. For example, high calcium levels within α7 nAChR and ER containing cellular microdmains that have been suggested in the growth cone [56] may drive the activation of the low calcium affinity type 2 calpain while broad calcium transients may drive calpain 1 in other parts of the cells. In future studies it will be interesting to discern specificity between α7 nAChR calcium signals and calpain isoform function.

### α7 nAChR signaling through calpain in axon growth

**Fig 9:**
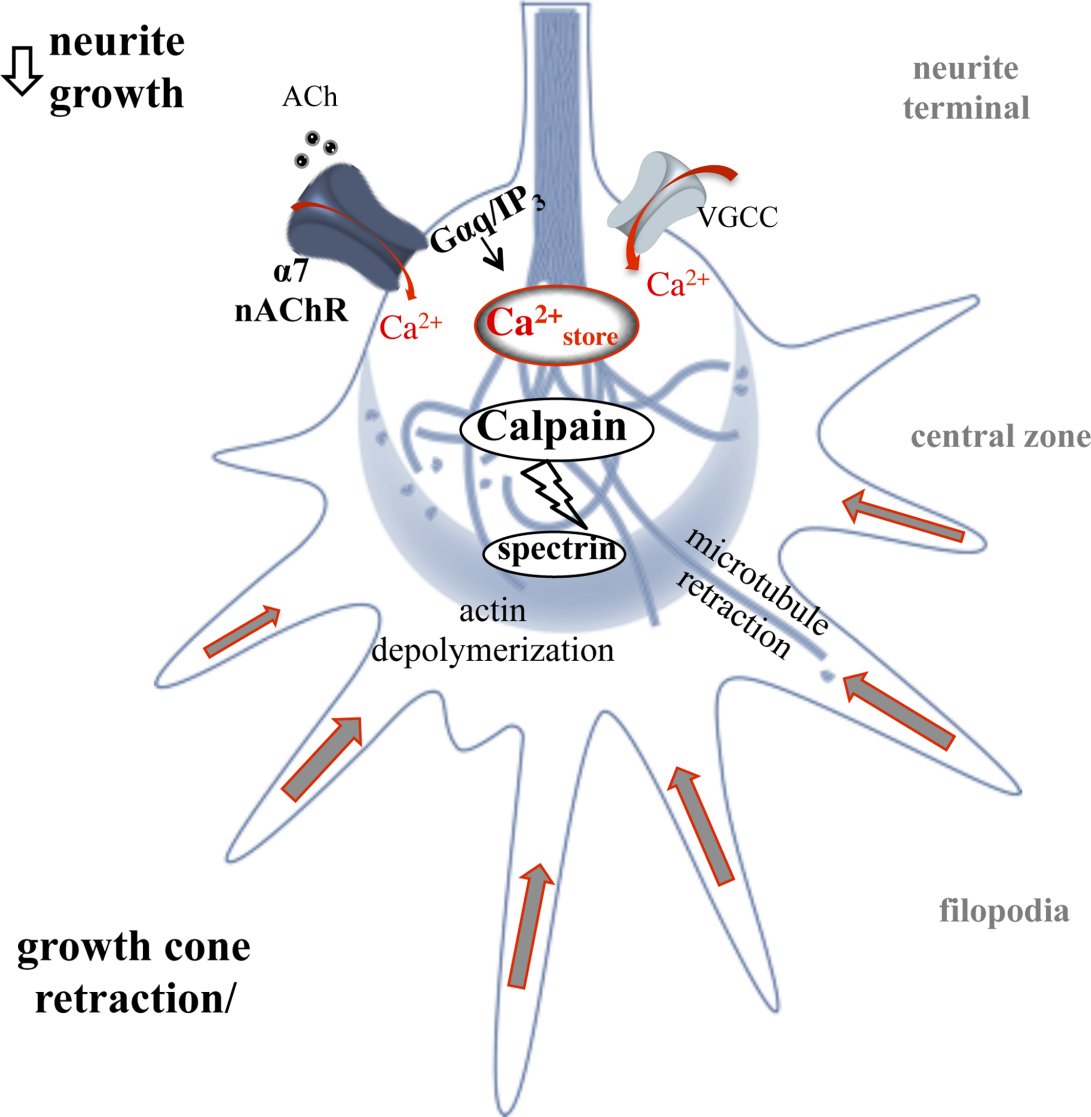
A schematic illustration of α7 nAChR mediated calpain activity in the growth cone. Ligand stimulation of the α7 nAChR (e.g. ACh) mediates a rise in local intracellular calcium via calcium influx leading to CICR and Gαq associated IICR leading to cytoskeletal breakdown through calpain. This process can drive rapid change in the growth cone and impact long term neurite growth.

Morphogenic cholinergic signals are well documented in embryonic development and contribute to structure and connectivity in the CNS [48]. A strong role for the α7 nAChR in the regulation of axon development through calcium signaling in the growth cone has been documented in various neuronal systems [4,57]. In fact, α7 nAChRs are selectively targeted to the growth cone compartment in neurons through binding the G protein scaffold molecule, G protein-regulated inducer of neurite outgrowth 1 [28]. Once there, α7 nAChRs operate through both ionotropic and metabotropic G protein signaling thereby activating various calcium signaling mechanisms [22,29]. This study extends these findings by showing a role for α7 nAChR calcium signaling in cytoskeletal breakdown at the growth cone through calpain (Fig 9). Co-localization of the α7 nAChR and the ER within the central zone appears important for calcium signaling as evidenced by previous findings that demonstrate that blockade of the IP_3_R receptor, or uncoupling from Gαq signaling, decreases the ability of α7 nAChRs to mediate calcium transients in the growth cone [20,22]. Based on experimental and computational findings, calcium levels produced by α7 interactions with the ER at the growth cone are sufficient for calcium activation of calpain and thus can directly drive spectrin breakdown locally [56]. Our findings support this model and show that pharmacological blockade of calcium release from the ER through the ryanodine receptor or the IP_3_R is sufficient to inhibit calpain function in the EB3 comet assay. In addition, metabtropic G protein signaling appears important for downstream calpain activation by the α7 nAChR as evidenced by the finding that expression of the α7_345-8A_ mutant or application of the Gαq blocker (Sub P) impairs the ability of choline to breakdown spectrin.

While a large number of α7 nAChRs are presynaptic this receptor is also expressed in somatodendritic regions and has been shown to regulate plasticity leading to long term potentiation [19,58,59]. Studies suggest that calpain function regulates structure and activity in postsyanptic compartments as well. For example, activation of calpain 2 is found to increase protein sysnthesis in the spine via the clevage of the phosphotase and tensin homolog (PTEN). In addition, calpain 1 has been shown to cleave RhoA leading to rapid cytoskeletal remodeling in dendritic spines during intracellular calcium transients [60,61]. Previously, we have shown a role for α7 nAChR calcium signlaing through RhoA in actin assembly at the growth cone [29] suggesting that similar mechanisms of calcium driven structural plasticity may exist in axons as well as dendrites. These findings should be confirmed in future studies in systems such as hippocampal neurons in order to determine the role of α7 nAChR/caplain interaction in synaptic development and plasticity.

## Supporting information

S1 File EB3 comet FRAP measurements. (XLSX)

S2 File PC12 neurite outgrowth measurements. (XLSX)
